# Cost-effectiveness of enzyme replacement therapy with alglucosidase alfa in classic-infantile patients with Pompe disease

**DOI:** 10.1186/1750-1172-9-75

**Published:** 2014-05-16

**Authors:** Tim A Kanters, Iris Hoogenboom-Plug, Maureen PMH Rutten-Van Mölken, W Ken Redekop, Ans T van der Ploeg, Leona Hakkaart

**Affiliations:** 1 Institute for Medical Technology Assessment, Erasmus University Rotterdam, Burgemeester Oudlaan 50, P.O. Box 1738, 3000DR Rotterdam, The Netherlands; 2 Center for Lysosomal and Metabolic Diseases, Erasmus MC University Medical Center, Dr. Molewaterplein 60, 3015 GJ Rotterdam, The Netherlands

**Keywords:** Pompe disease, Infants, Enzyme replacement therapy, Cost-effectiveness, Quality adjusted lifeyears

## Abstract

**Background:**

Infantile Pompe disease is a rare metabolic disease. Patients generally do not survive the first year of life. Enzyme replacement therapy (ERT) has proven to have substantial effects on survival in infantile Pompe disease. However, the costs of therapy are very high. In this paper, we assess the cost-effectiveness of enzyme replacement therapy in infantile Pompe disease.

**Methods:**

A patient simulation model was used to compare costs and effects of ERT with costs of effects of supportive therapy (ST). The model was filled with data on survival, quality of life and costs. For both arms of the model, data on survival were obtained from international literature. In addition, survival as observed among 20 classic-infantile Dutch patients, who all received ERT, was used. Quality of life was measured using the EQ-5D and assumed to be the same in both treatment groups. Costs included the costs of ERT (which depend on a child’s weight), infusions, costs of other health care utilization, and informal care. A lifetime time horizon was used, with 6-month time cycles.

**Results:**

Life expectancy was significantly longer in the ERT group than in the ST group. On average, ST receiving patients were modelled not to survive the first half year of life; whereas the life expectancy in the ERT patients was modelled to be almost 14 years. Lifetime incremental QALYs were 6.8. Incremental costs were estimated to be € 7.0 million, which primarily consisted of treatment costs (95%). The incremental costs per QALY were estimated to be € 1.0 million (range sensitivity analyses: € 0.3 million - € 1.3 million). The incremental cost per life year gained was estimated to be € 0.5 million.

**Conclusions:**

The incremental costs per QALY ratio is far above the conventional threshold values. Results from univariate and probabilistic sensitivity analyses showed the robustness of the results.

## Introduction

Since the introduction of orphan drug regulations, the number of orphan drugs (i.e. drugs for rare diseases) has grown vastly. This confronts policy makers with a trade-off between access and affordability. On the one hand, the overall proportion of orphan drugs in health care expenditures is substantial and continues to grow [1]. On the other hand, healthcare authorities would like to provide rapid access to promising new treatments, even when the evidence base might not be mature yet. To deal with this trade-off, policy makers can turn to ‘coverage with evidence development schemes’, which enable patients to obtain access to the treatment while effectiveness is simultaneously studied in a real-world setting [2,3]. When a cost-effectiveness analysis is performed at the same time, policy makers also gain insight in the economic consequences of the new treatment. In 2006, such a scheme was installed in the Netherlands for high-priced in-hospital orphan drugs. During a coverage with evidence development period of four years, effectiveness and cost-effectiveness were studied for orphan drugs listed on a specific policy rule [4].

One of the drugs reimbursed through this policy rule is a drug to treat Pompe disease. Pompe disease is a rare metabolic disease and presents as a broad clinical spectrum, with the rapidly progressive classic-infantile form at the most severe end and late-onset or adult-onset Pompe disease at the least severe end [5,6]. In all cases, the disease is caused by a deficiency of the enzyme acid α-glucosidase. The incidence of classic-infantile Pompe disease is 1 in 138,000 births [7]. In classic-infantile Pompe disease, symptoms present in the first months of life and involve respiratory and feeding problems, airway infections, and generalized muscle weakness. Patients also show progressive thickening of the heart (hypertrophic cardiomyopathy) which eventually leads to heart failure. These children generally die before the first year of age from cardiorespiratory failure and the median age of death has been estimated to be 6 to 9 months [8,9].

Enzyme replacement therapy (ERT) with alglucosidase alfa (Myozyme®, Genzyme corp.) was developed as a treatment for Pompe disease. ERT has proven to have a substantial effect on survival in classic-infantile Pompe patients, reducing the 3-year mortality risk by 95% compared to an untreated historical control group [10,11]. Cardiac, respiratory and motor functions of patients have been shown to improve by therapy.

Orphan drugs are often very expensive and this is also true for ERT to treat Pompe disease [12]. As of 2006, ERT for Pompe disease is reimbursed in the Netherlands under a coverage with evidence development scheme, during which the cost-effectiveness of the treatment needs to be assessed, even though this was expected to be unfavorable upfront. This study reports on the cost-effectiveness of ERT in classic-infantile Pompe disease.

## Methods

### Patients and treatments

All Dutch patients with classic-infantile Pompe disease were enrolled in an observational study. Diagnosis of Pompe disease was confirmed by enzyme assay in leukocytes or fibroblasts and/or mutation analysis. All patients were treated by the Erasmus MC Center for Lysosomal and Metabolic Diseases, Rotterdam, the Netherlands. The Institutional Review Board approved the studies. Written informed consent was provided by parents.

Currently ERT is the only available registered treatment for Pompe disease. In this study, the comparative treatment therefore consisted of usual supportive therapy (ST), including for example (nightly) ventilation, surgical correction of scoliosis, or nutritional support.

The registered dose of ERT is 20 mg/kg/2 weeks. Patients in the Netherlands received doses ranging from 20 mg/kg/2 weeks to 40 mg/kg/week. Since 2008 the majority of patients use the higher dose. The majority of the Dutch data was collected in patients using this dose. In the base case analyses of the model we therefore used the maximum dose of 40 mg/kg/week.

### Study design and model structure

A patient simulation model was used to compare costs and effects of ERT with costs and effects of ST for patients with classic-infantile Pompe disease. The model was filled with data on survival, utilities and costs. For both treatment arms, the model generated costs, survival, quality of life and quality adjusted life years (QALYs). In addition, the model generated an estimate of the cost-effectiveness of treatment, expressed as cost per QALY.

### Effects

#### Survival

Survival for the ST cohort was retrieved from two international studies on the natural course of infantile Pompe disease (n = 172; maximum 24 months follow-up [9] and 119 cases from literature [8]). Survival for the ERT cohort was obtained from three sources to increase sample size, i.e. a trial extension study (n = 18; maximum 36 months follow-up) [11], an international open-label study (n = 21; median follow-up 28 months) [13] and data from Dutch infantile patients under treatment at Erasmus MC (n = 20; median follow-up 32 months). For all cohorts, patient-level data was available to include in the survival analysis. Table 1 provides characteristics of the patients in the three cohorts; the proportion of patients that died and used ventilation differed between cohorts, as did the dosage regimens. To extrapolate survival beyond the observed period, parametric survival models were fitted. Several distributions were investigated (exponential, Weibull, lognormal, and loglogistic). The choice of distribution was based on visual inspection and fit of the model to the data according to Akaike Information Criterion (AIC) and Bayesian Information Criterion BIC) [14]. When predicted survival was higher than in the general population, we have applied the mortality rates of the Dutch population [15].

**Table 1:** Patient characteristics for patients in survival analyses

	**Kishnani (2009)**	**Nicolino (2009)**	**Erasmus MC**
N	18	21	20
Deaths	5 (28%)	6 (29%)	4 (20%)
Age at end study in months [range]	34.5 [19.7-44.0]	41.0 [7.7-80.3]	60.9 [3.2-178.8]
Patients using ventilation	9 (50%)	7 (33%)	5 (25%)^b^
Dosage (every other week)			
15 mg/kg	0	0	2^c, d^
20 mg/kg	9	21^a^	8^e^
40 mg/kg	9	0	10^d^

^a^Eight patients switched to 40 mg/kg after 26 weeks due to clinical deterioration; ^b^Two patients died; ^c^Both patients switched to 30 mg/kg and later to 40 mg/kg; ^d^Every week; ^e^Five patients switched to 40 mg/kg.

#### Quality of life

Quality of life was assessed in Dutch patients using the Euroqol-5D (EQ-5D), completed by parents of patients every six months. The EQ-5D is a validated instrument for measuring and valuing generic health related quality of life [16]. The instrument describes 245 health states, and each health state is associated with a utility using a scoring formula. Utility scores typically range from zero (death) to 1 (perfect health). Utility scores were estimated using the Dutch tariff [17]. Only observations for patients above the age of two years were included (n = 6; median follow-up 24 months). The average utility was 0.62, ranging from 0.24 to 0.82. For five patients, multiple observations were available; their average utility was used in the analyses.

#### Costs

Costs were calculated from a societal perspective. This implies that all costs are included, no matter to whom they accrue. Total costs for patients treated with ERT consisted of four components: the cost of the drug alglucosidase alfa, infusion-related costs, costs related to other health care use and informal care costs. Patients receiving ST did not incur costs of the drug and infusion-related costs. Costs were expressed in 2009 euro values.

### Treatment costs

Costs of the drug alglucosidase alfa are dependent on patient’s weight. In the Netherlands, costs per vial (50 mg) are €556.50. In the Netherlands the doses applied in infants with classic-infantile Pompe disease range from 20 mg/kg/2 weeks to 40 mg/kg/week bodyweight (since 2008 the majority of patients use the higher dose). For the model we used the maximum dose of 40 mg/kg/week so drug costs per kilo bodyweight were €445.20. With weekly infusions (52 infusions per year), yearly medication costs per kilo are €445.20*52 = €23,150.40. Data from the Dutch cohort were collected between May 2007 and October 2012. Patients’ weights were estimated on the basis of available data on Dutch patients (n = 17; median follow-up 35 months), and increased with the patient’s age to a maximum of 75 kilograms.

Infusion-related costs were based on detailed time studies using the methodology described in the Dutch costing manual [18]. The total costs for infusion consisted of cost associated to physician and nursing time during infusion, overhead, capital, materials, informal care and travel time. A distinction was made with respect to treatment location of patients; patients receive infusions at home or in hospital. The estimated mean cost per infusion at home was € 426 compared to € 520 per infusion at the hospital. A total percentage of 68% of the Dutch patients were treated in the home situation. Based on weekly infusions (52 infusions per year), the annual infusion costs were estimated to be € 23.710.

#### Other costs

Data of other health care utilization were collected by means of a health economic questionnaire, completed by the parents of the Dutch patients (n = 12; median follow-up 11 months). Bottom-up methodology was used to calculate the total direct medical costs; that is, the total number of physician and other caregiver contacts multiplied by unit costs of the corresponding health care services. Reference unit prices of health care services from the Dutch costing manual [18] were applied. Costs of informal care were valued using the shadow price method, also following the costing manual. The estimation of health care costs was described in more detail for adult non-treated patients [19]; for infantile patients the same methodology was used. Table 2 provides the unit costs used. Due to their age, infantile patients did not incur indirect costs from productivity losses.

**Table 2:** Cost components and associated unit costs (2009 prices)

**Cost component**	**Cost per unit**^**b**^	**Source**
Hospital day		
Regular ward	€ 394^a^	[18]
Intensive care	€ 1,847	[18]
Ambulatory care		
Hospital day visits	€ 69^a^	[18]
General practitioner visit	€ 22	[18]
Physiotherapy	€ 25	[18]
Other paramedical	€ 14 - € 91	[18]
Home care per hour	€ 29 - € 65	[18]
Medication		[20]
Other medical costs		
Tests & procedures	€ 54 - € 181	[18]
Respiratory support per day	€ 5	[21]
Medical devices	€ 18 - € 1,500	Market prices
Informal care costs per hour	€ 9	[18]

^a^weighted average of academic and general hospital costs ^b^costs per unit are based on average unit costs for medical procedures, consultations and admissions [18].

Other health care utilization costs were estimated using a generalized estimated equation (GEE) model, a logarithmic link function and a gamma distribution (n = 12). Age was the only predictor variable used in this model.

#### Model assumptions

No data was available on health related quality of life (utility) for patients receiving ST. Utilities were therefore assumed to be equal in the two treatment arms. Hence, differences in QALYs only resulted from differences in life expectancy between the two treatment arms. Health care utilization costs were only available for patients receiving ERT. We assumed that patients receiving ST incurred the same costs as ERT-treated patients with the exception of treatment costs. Differences in costs therefore only resulted from treatment costs and differences in life expectancy between the two treatment arms.

#### Analyses

Cost-effectiveness was expressed in incremental cost per QALY gained and incremental cost per life year gained. Costs were discounted at a rate of 4% and effects were discounted at a rate of 1.5% in accordance with Dutch guidelines for pharmacoeconomic research [22]. A lifetime time horizon was used and a cycle length of ½ year was used.

Univariate sensitivity analyses were performed to examine the impact of the assumptions of the model on the results. We varied the following input variables: ERT dosage (the registered dose of 20 mg/kg/2 weeks as opposed to the mostly used dose in the Netherlands of 40 mg/kg/week in the base case analysis); costs of treatment (€ 11,575.20 per kilo per infusion as opposed to € 23,150.40 per kilo per infusion); time horizon (5 years as opposed to lifetime in the base case); quality of life (0.49 and 0.74 in both cohorts and 0.31 in the ST cohort combined with 0.62 in the ERT cohort – implying a treatment effect on quality of life – as opposed to 0.62 in both cohorts); survival in the ERT cohort (varying the distributions used in the parametric survival analyses); and costs incurred by the ST cohort (double costs and no costs for the ST-cohort instead of assuming that ‘other healthcare costs’ were the same as the costs seen in ERT-treated patients).

Next to the univariate sensitivity analyses, probabilistic sensitivity analyses were performed to examine the impact of the uncertainty around the values of the input variables on the estimated effectiveness and cost-effectiveness of ERT. For this purpose, 1,000 populations were randomly drawn from relevant distributions in a Monte Carlo simulation procedure. The results from the probabilistic sensitivity analyses were presented in a cost-effectiveness plane (CE-plane). The CE-plane shows total incremental costs and total incremental effects of ERT against ST.

## Results

### Patient characteristics

Estimates for health care utilization costs were based on data of 12 Dutch classic-infantile patients; 8 male patients and 4 female patients. At first measurement, average age of these patients was 3.5 years. Data on quality of life was available for six patients; four male patients, average age 6.1 years (range 2.2 – 11.1) at baseline.

### Effects

**Figure 1:**
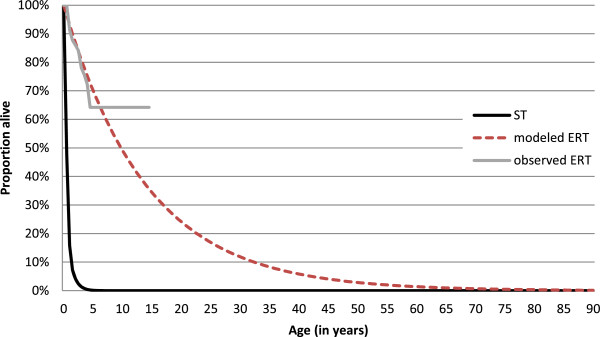
Observed and modeled survival curves.

Figure 1 provides observed and modeled survival in both cohorts. On average, ST-treated patients did not survive the first half year of life (mean life expectancy 0.40 years), depicted by the steep decline of the solid black curve in Figure 1. Life expectancy was considerably longer in the ERT cohort. The exponential survival function best fitted the data for ERT-treated patients and is depicted in Figure 1 by the dashed line. The modeled survival followed the observed survival very closely in the first four years. From the age of 5 years, no deaths were observed in the ERT-group (solid grey curve) and the observed ERT-curve becomes flat accordingly. For this period, observed survival is heavily influenced by a small number of patients with a long follow-up. The observed and modeled survival curves for the ERT-group diverge from this point on. Table 3 shows the results for the total and incremental effects. The life expectancy in the ERT cohort was estimated to be 13.8 years (mean QALYs: 7.00). Lifetime incremental QALYs were estimated to be 6.75 (7.00 vs. 0.24). Effects were discounted at a rate of 1.5% per year in both cohorts. For the ERT cohort the influence of discounting is larger, as effects occur over a longer time span, than for the ST cohort.

**Table 3:** Total and incremental costs and effects

	**ST**	**ERT**	**Difference**	**[95% confidence interval]**
Life expectancy (years)	0.40	13.79	13.39	[1.55 – 25.23]
QALYs	0.24	7.00	6.75	[2.32 – 11.19]
Total costs	€ 32,871	€ 7,032,899	€ 7,000,028	[1,869,635 – 12,130,422]
- ERT costs	€ -	€ 6,630,525	€ 6,630,525	[1,615,059 – 11,645,991]
- Infusion costs	€ -	€ 212,793	€ 212,793	[121,477 – 304,108]
- Other costs	€ 32,871	€ 189,582	€ 156,711	[131,728 – 181,694]

*ST* supportive therapy, *ERT* enzyme replacement therapy, *QALY* quality adjusted life year.

### Costs

Table 3 also shows the total and incremental costs for classic-infantile patients. The majority of the incremental costs consisted of drug costs (95% of incremental costs). In addition, infusion costs were estimated to be € 212,793 (3.0%). ERT-treated patients incurred higher costs than ST-treated patients not simply because of the ERT treatment they received but also because they lived much longer. Incremental costs were estimated to be € 7.0 million.

### Cost-effectiveness

Table 4 shows the incremental cost-effectiveness ratio for the treatment of classic-infantile Pompe patients with ERT. The incremental costs per QALY were estimated to be € 1.0 million. The incremental cost per life year gained was estimated to be € 0.5 million.

**Table 4:** Incremental cost-effectiveness ratios (dosage 40 mg/kg/week unless otherwise specified)

	**Costs/QALY gained**	**Costs/life year gained**
Base case analysis dosage 40 mg/kg/week	€ 1,043,868	€ 525,873
Registered dosage regimen (20 mg/kg/2 weeks)	€ 286,114	€ 144,137
Lower treatment costs (costs divided by 2)	€ 549,280	€ 276,713
Shorter time horizon (5 years)	€ 571,701	€ 92,634
Lower utility (0.49) in both cohorts	€ 1,304,835	€ 525,873
Higher utility (0.74) in both cohorts	€ 869,890	€ 525,873
Lower utility in ST-treated patients	€ 1,021,610	€ 525,873
Lognormal survival distribution	€ 1,050,595	€ 452,669
No costs incurred by ST-treated patients	€ 1,049,203	€ 528,560
Double cost incurred by ST-treated patients	€ 1,031,836	€ 520,387

### Sensitivity analyses

Table 4 also provides the results from the sensitivity analyses. The first sensitivity analysis examined the effect of dosage on the ICER. Total costs for ERT-treated patients were considerably lower in the 20 mg/kg/2 weeks analysis than in the base case analysis, because less medication was administered. Incremental costs were estimated to be € 1.9 million. The ICERs at the lower, biweekly dose were about 3.6 times lower than the ICER in the base case analysis.

In a sensitivity analysis, the influence of treatment costs on the ICER was examined by halving these costs. The ICER dropped substantially (47%), indicating the prominent role of treatment costs in the analyses.

When a shorter time horizon of 5 years was used, the ICER was lower relative to the analyses with a lifetime horizon. During this period, patients’ body weights’ were relatively low, which considerably decreased treatment costs leading to more favorable ICERs. Incremental costs in this analysis were estimated to be € 1.2 million and the incremental effects to be 2.20 QALYs.

Since information on utility (quality of life) was limited, we varied the utility value used in the base-case analysis by 20% to determine how much it affected the results (range: 0.49 to 0.74). A change in quality of life can only affect QALY gain and thereby the ICER; it has no impact on the incremental costs. A lower value for quality of life reduced QALY gains and led to an increase in the ICER. When a higher utility value was used, the QALY gain increased and the ICER decreased. The use of a lower utility in the ST-treated patients only (implying a treatment effect on utility) did not change the ICER substantially.

Survival in the ERT-treated patients was based on an exponential survival distribution, because that fitted the data best. Use of the lognormal distribution, which had the second lowest BIC, increased life expectancy from 13.8 to 21.9 years, primarily because the predicted survival later in life was higher. Incremental QALYs increased to 9.3 in this analysis. This longer life expectancy increased the incremental costs to 9.7 million. However, this did not affect the ICER, which remained 1.04 million/QALY. Two other distributions were also tested (Weibull, log-logistic), but they did not have any substantial effect on the ICER (Weibull: life expectancy 14.9 years; ICER 1.05 million/QALY; log-logistic distribution: life expectancy 20.6 years; ICER 1.03 million/QALY).

Since no data was available regarding costs of ST treatment we assumed in the base case analysis that these costs were the same as the costs seen in the ERT-treated group (excluding ERT-related costs). We tested the importance of this assumption by doubling the costs for the ST cohort and by setting the total costs in the ST cohort to zero. However, since the costs for the ST cohort in the base case analysis were limited because of the short life expectancy in this group, changes in the costs for the ST cohort had no appreciable influence on the ICER.

### Probabilistic sensitivity analyses

**Figure 2:**
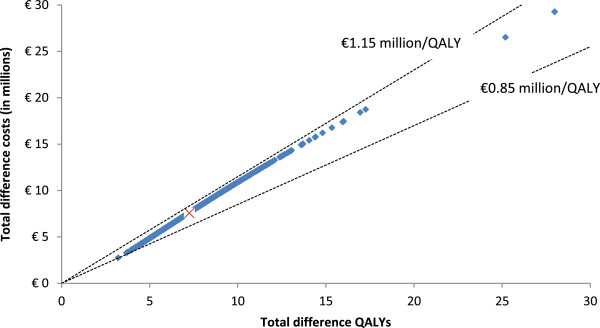
Cost-effectiveness plane: incremental costs and incremental effects of enzyme replacement therapy relative to supportive therapy.

Figure 2 provides the results from the probabilistic sensitivity analyses in a cost-effectiveness plane (CE-plane). All outcomes of the probabilistic sensitivity analyses are in the northeast quadrant of the CE-plane, i.e. all draws resulted in better health outcomes and higher costs. Furthermore, the CE-plane shows a strong positive association between incremental costs and effects. The CE-plane further shows that the dispersion of incremental costs and effects from the average incremental costs and effects (depicted by the X in the CE-plane) is quite large. However, the variation of the ICERs is very limited; all estimates are within a range of €0.85 million/QALY and €1.15 million/QALY.

## Discussion

This is one of the first studies to assess the cost-effectiveness of an orphan drug, evaluating the cost-effectiveness of enzyme replacement therapy with alglucosidase alfa (Myozyme®) in classic-infantile Pompe disease. The incremental cost-effectiveness was calculated on the basis of available data, a pharmacoeconomic model and assumptions on disease course. The cost per QALY was estimated to be € 1.0 million; cost per life year gained was € 0.5 million.

The results from the univariate sensitivity analyses and probabilistic sensitivity analyses showed the robustness of the model. Uncertainty with regard to the ICER is limited; in all cases the ICER is beyond any conventionally used cost-effectiveness threshold. While the absolute gains in life years, incremental QALYs, and incremental costs differ between various sensitivity analyses, ICERs are not affected.

For instance, using log-normal and log-logistic distributions to estimate survival had a large impact on the life expectancy. This was primarily caused by a higher survival later in life. However, despite the large gains in survival, the ICER was relatively unaffected, due to the significant costs of therapy. This shows the paradoxical situation of performing an economic evaluation with a therapy of such high annual costs; better effectiveness did not lead to a better cost-effectiveness ratio.

This study was performed using currently available information, but the available evidence on (infantile) Pompe disease is increasing as the follow-up period increases. For instance, some patients have already survived to the age of 15 and hope to reach adulthood. Increases in the number of patients and follow-up time will lead to more stable estimates of survival.

### Limitations of the study

There are a number of limitations of the study that need to be stressed. Most limitations are due to the relatively scarce availability of data, both with respect to number of patients (due to orphan disease status) and time period involved.

Firstly, survival estimates from various sources were combined to increase sample size. Hence, the implicit assumption was that survival probabilities for patients were comparable, although the doses used in these groups of patients varied. The number of patients was too small to perform subgroup analysis by dosage. Furthermore, survival was modeled using an exponential survival distribution, which assumes a constant hazard over time. The choice of the distribution was made on the basis of visual inspection and best fit of the data [14]. Although a constant hazard might not be a realistic assumption, the sensitivity analyses showed that the choice of the distribution did not have a large influence on the ICER.

Secondly, the pharmacoeconomic model was based on observations of a limited period. We assumed that these results could be extrapolated into the future. Accordingly, we assumed that patients did not change therapy over the course of time. In addition, costs and effectiveness of the treatment were assumed not to change.

A third point of attention concerns the valuation of health related quality of life, which was assessed using a proxy version of the EQ-5D. The use of a proxy to make statements on a subjective measure as quality of life can be difficult. However, for young children alternatives are limited [23]. In addition, we only used the utility observations of children older than two years of age to estimate the utility of the entire group, because of reasons of applicability of the EQ-5D items. The assumption of equal utilities in both treatment arms was made due to these data constraints, and represented a conservative scenario. Sensitivity analyses showed that the utility level in the ST-cohort did not influence the results.

Finally, in the current model we used a base case in which patients received 40 mg/kg/week, since this is the dosage regimen used by the majority of Dutch patients. We also used information on patients receiving other dosages (particularly 20 mg/kg/2 weeks) to build the model. For these patients, treatment costs are lower and effects are likely to be lower. In the sensitivity analyses, the effect of dosage on costs was examined, keeping effectiveness constant. A lower dosage and fewer infusions reduced the ICER substantially. However, it is likely that a lower dosage also leads to a reduction in effects. In that case, the ICER would increase.

### Future research

The current study assesses the cost-effectiveness of ERT in Pompe disease only in the severe infantile form of the disease; results may differ for other populations.

A lively debate has taken place in the literature as to whether or not orphan drugs should be excluded from any cost-effectiveness assessment [24,25]. The most prominent question is whether society is willing to pay a premium because of the rarity of a disease. A recent study showed that this might not be the case, at least in Norway [26]. In contrast, the Dutch Health Care Insurance Board seems to place extra value on rarity, judging by the advice to the Minister of Health to reimburse Myozyme® in infantile Pompe disease in 2012. This decision is probably also driven by the relatively small budget impact. Other factors thus play a role in reimbursement decisions. This hints at the potential role for multi-criteria decision analyses in reimbursement decisions of orphan drugs, although deriving weights for the different criteria might be a major challenge.

## Conclusions

In this study, the cost-effectiveness of enzyme replacement therapy with Myozyme® in classic-infantile Pompe disease was assessed. Incremental costs per QALY were estimated to be € 1.0 million.

## Competing interest

The study was financially supported by the Netherlands Organization for Health Research and Development (ZonMw; grant number 152001005). Since August 2004, A.T. vd.P. has provided consulting services for Genzyme Corp, Cambridge, MA, USA, under an agreement between Genzyme Corporation and Erasmus MC, Rotterdam, the Netherlands. This agreement also covers financial support for Erasmus MC to pursue research in the field of Pompe disease. Erasmus MC and inventors of the method of treatment of Pompe disease by enzyme replacement therapy receive royalty payments pursuant to Erasmus MC policy on inventions, patents and technology transfer.

## Authors’ contributions

TK performed the statistical analyses and drafted the manuscript. All authors participated in the study design, contributed to the interpretation of the results and revised the manuscript. All authors read and approved the final manuscript.
